# Projection-Related Bias in the Detection of Thoracic Abnormalities: A Large-Scale Analysis of the NIH ChestX-Ray14 Dataset

**DOI:** 10.3390/jimaging12050187

**Published:** 2026-04-27

**Authors:** Josef Yayan

**Affiliations:** Department of Internal Medicine, Division of Pulmonary, Allergy, and Sleep Medicine, Helios University Hospital Wuppertal, Witten/Herdecke University, Heusnerstr. 40, 42283 Wuppertal, Germany; josef.yayan@hotmail.com; Tel.: +49-0202-896-3936; Fax: +49-0202-896-3901

**Keywords:** chest radiography, projection bias, anteroposterior projection, posteroanterior projection, artificial intelligence, dataset bias

## Abstract

Chest radiography remains a cornerstone in the diagnosis of thoracic diseases. However, differences in image acquisition—particularly projection type—may influence the apparent prevalence and detectability of radiographic findings. Such differences may represent a potential source of bias in large imaging datasets used for clinical research and artificial intelligence. Importantly, projection type is closely associated with the patient’s condition and may therefore reflect both technical imaging factors and underlying clinical characteristics, including disease severity. A total of 120,120 chest radiographs were available in the dataset. After applying inclusion criteria, 112,104 images were included in the primary analysis. Multivariable logistic regression models were used to assess the association between projection type and the presence of radiographic findings, adjusted for age and sex. Subgroup and interaction analyses were performed to evaluate effect modification by demographic factors. Given the large sample size, emphasis was placed on effect sizes and confidence intervals rather than statistical significance alone. Compared with posteroanterior projection, anteroposterior projection was associated with higher odds of detecting consolidation (aOR 3.27; 95% CI 3.07–3.48), infiltration (aOR 1.90; 95% CI 1.84–1.96), pleural effusion (aOR 1.66; 95% CI 1.60–1.72), atelectasis (aOR 1.63; 95% CI 1.57–1.70), and cardiomegaly (aOR 1.19; 95% CI 1.10–1.28). These associations were consistent across age and sex strata. A significant interaction between projection type and sex was observed for infiltration (*p* = 0.01). Projection type is associated with substantial differences in the detection of thoracic abnormalities on chest radiographs. These associations should be interpreted with caution, as they likely reflect a combination of technical imaging effects and residual confounding related to patient severity and clinical context. Projection may therefore act as a marker of dataset heterogeneity rather than a purely causal factor. Accounting for projection metadata is therefore essential to improve clinical interpretation and to ensure the robust development and validation of artificial intelligence models.

## 1. Introduction

Frontal chest radiography is one of the most frequently performed imaging examinations worldwide and plays a central role in the evaluation of pulmonary and cardiothoracic diseases. Depending on the clinical setting and the patient’s condition, chest radiographs are typically acquired in either posteroanterior (PA) or anteroposterior (AP) projection. PA imaging is generally preferred in ambulatory patients, whereas AP projection is commonly used in immobile or critically ill individuals, particularly in emergency departments and intensive care units [1,2].

With the increasing use of large imaging datasets for clinical research and artificial intelligence development, systematic differences in image acquisition have gained growing attention. Among these, projection type represents a key technical parameter that may influence both image appearance and diagnostic labeling. Compared with PA views, AP projections often exhibit lower image resolution, magnification of the cardiac silhouette, and altered visualization of pulmonary structures [3,4]. These differences may affect diagnostic interpretation and introduce systematic variability in the reported prevalence of radiographic findings.

Large publicly available imaging datasets, such as the NIH ChestX-ray14 dataset, provide a valuable opportunity to investigate how acquisition-related factors influence radiographic labels at scale [5]. In addition, other large-scale datasets such as the CheXpert dataset have highlighted challenges related to dataset composition, label uncertainty, and acquisition heterogeneity in medical imaging research [5]. However, because projection type is closely linked to clinical context—such as patient mobility and disease severity—it may also act as a proxy for underlying patient characteristics. In particular, AP projection is frequently associated with higher patient acuity, introducing the potential for substantial confounding by indication. As a result, observed differences in radiographic findings between AP and PA projections may reflect both technical imaging effects and differences in patient populations.

A previous study using the same dataset demonstrated projection-related differences in the prevalence of selected thoracic abnormalities, including atelectasis, pleural effusion, and pulmonary nodules [6]. However, the extent to which these associations generalize across additional findings and patient subgroups remains incompletely characterized. The present study extends these findings by evaluating additional radiographic patterns, including infiltration, consolidation, and cardiomegaly, and by examining projection-related variability across demographic subgroups.

This study aims to quantify the association between projection type and multiple thoracic abnormalities in a large-scale dataset, assess the consistency of these associations across age and sex subgroups, and evaluate projection type as a potential marker of both technical imaging differences and underlying patient-related confounding relevant to clinical interpretation and artificial intelligence applications.

Given the observational design, this analysis does not aim to establish causal relationships but rather to characterize systematic associations that may arise from both imaging-related and clinical factors. In addition, we assessed whether these associations varied across age groups and between sexes and whether they remained robust after adjustment for available covariates. By explicitly addressing potential sources of bias and confounding, this study provides a cautious interpretation of projection-related variability and its implications for clinical research and the development of artificial intelligence models.

## 2. Materials and Methods

### 2.1. Study Design and Dataset

This retrospective observational study was conducted using the publicly available NIH ChestX-ray14 dataset, an expanded version of the original ChestX-ray8 dataset [5,7]. The dataset comprises 120,120 chest radiographs obtained from 32,717 unique patients and includes labels for common thoracic findings generated using natural language processing (NLP) applied to radiology reports by the dataset developers [5,7].

Each image is accompanied by metadata including patient age, sex, and radiographic projection type. For the present analysis, only frontal chest radiographs with clearly defined projection labels (AP or PA) and complete demographic information were included. Lateral images and radiographs with missing or ambiguous projection data were excluded. After applying these inclusion criteria, a total of 112,104 radiographs were included in the primary analysis, comprising 67,299 PA and 44,805 AP images.

### 2.2. Variables and Definitions

The primary independent variable was projection type, categorized as AP versus PA. Dependent variables included the presence or absence of the following radiographic findings: infiltration, consolidation, pleural effusion, atelectasis, and cardiomegaly. These labels were obtained directly from the ChestX-ray14 dataset annotations derived using NLP techniques.

It is important to note that NLP-derived labels do not represent ground truth and may be subject to misclassification, as previously reported for large chest radiograph datasets, particularly for findings such as infiltration and consolidation [7]. In addition, differences in radiology reporting practices between AP and PA examinations may introduce differential misclassification, which could influence the observed associations.

A composite endpoint, referred to as “pneumonia-like”, was defined as the presence of either infiltration or consolidation. This composite outcome was included for exploratory purposes only and should be interpreted cautiously, as it does not correspond to a clinically validated definition of pneumonia.

Age (in years) and sex (male or female) were included as covariates in all regression models. No direct measures of disease severity or clinical context were available in the dataset. Where available, pixel spacing was included in sensitivity analyses to account for potential differences in image resolution.

### 2.3. Descriptive Analysis

Baseline characteristics were summarized separately for AP and PA projection groups. Continuous variables were reported as mean ± standard deviation (SD), median with interquartile range (IQR), and range (minimum–maximum). Categorical variables were expressed as counts and percentages. The distribution of age across projection types was visualized using histograms.

### 2.4. Statistical Analysis

The association between projection type and each radiographic finding was assessed using multivariable logistic regression models. All models were adjusted for age and sex. Results are presented as adjusted odds ratios (aOR) with 95% confidence intervals (CI).

Given the large sample size, emphasis was placed on effect sizes and confidence intervals rather than on *p*-values alone, as even small differences may reach statistical significance in large datasets. A two-sided *p*-value < 0.05 was considered statistically significant. Because multiple regression models were estimated, the potential for multiple hypothesis testing was considered, and a Bonferroni correction was applied (adjusted significance level α = 0.01).

All statistical analyses were performed using R software (R Foundation for Statistical Computing, Vienna, Austria, version 4.3.2). Regression models were implemented using the stats package, and graphical visualizations were generated using the ggplot2 package.

### 2.5. Subgroup and Sensitivity Analyses

Subgroup analyses were performed to evaluate the consistency of the association between projection type and pulmonary infiltration across age categories (<30, 30–49, 50–69, and ≥70 years) and by sex. To assess potential effect modification, an interaction term between projection type and sex (projection × sex) was included in the regression model.

Sensitivity analyses included adjustment for pixel spacing as an additional covariate where available. In addition, the results were interpreted in light of potential residual confounding due to unmeasured clinical variables, particularly disease severity and the patient’s condition.

### 2.6. Methodological Considerations

Because the ChestX-ray14 dataset contains multiple radiographs from individual patients, observations may not be fully independent. This lack of independence may lead to underestimated standard errors and overconfident effect estimates. Patient-level identifiers required for hierarchical or mixed-effects modeling were not consistently available, precluding formal adjustment for within-patient clustering.

Furthermore, the observational design and limited covariate availability restrict the ability to control for confounding by indication, particularly the known association between AP projection and higher patient acuity.

Radiographic labels were generated using automated NLP techniques applied to radiology reports and may therefore be subject to labeling inaccuracies. Previous validation studies have demonstrated variable label performance in large chest radiograph datasets, and the potential for both non-differential and differential misclassification should be considered when interpreting the results [7].

## 3. Results

**Figure 1:**
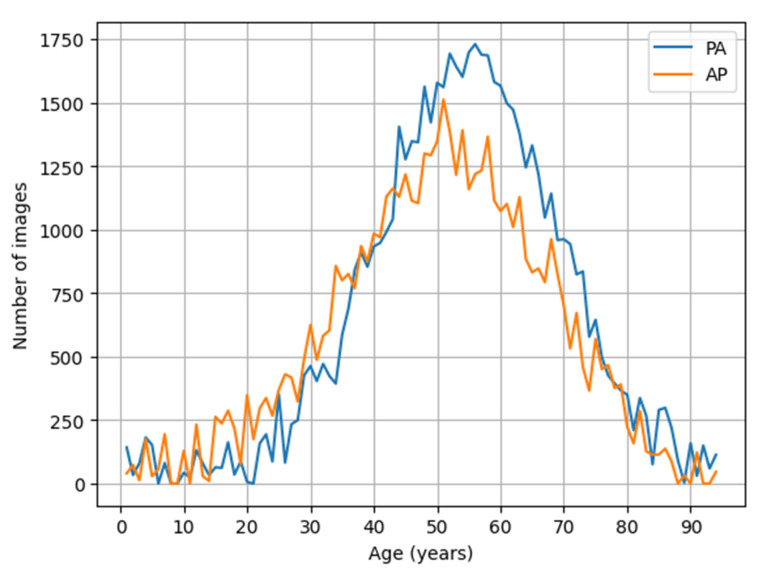
Age distribution of chest radiographs stratified by projection type (PA vs. AP). The number of chest radiographs is plotted across patient age for posteroanterior (PA, blue) and anteroposterior (AP, red) projections. To improve readability, age values are grouped and axis labels are spaced at regular intervals. PA images are more frequent in younger and middle-aged patients, whereas AP projections are more common in older age groups.

A total of 112,104 chest radiographs met the inclusion criteria and were included in the primary analysis, comprising 67,299 (60.0%) posteroanterior (PA) and 44,805 (40.0%) anteroposterior (AP) projections (Table 1). The mean age was comparable between the projection groups (PA: 47.3 ± 16.2 years; AP: 46.2 ± 17.2 years). AP projections were more frequently observed in older age groups, whereas PA projections predominated in younger and middle-aged individuals (Figure 1). The proportion of male patients was slightly higher in the AP group (58.5%) compared with the PA group (55.2%).

Multivariable logistic regression analyses adjusted for age and sex demonstrated an association between projection type and the detection of radiographic abnormalities (Table 2).

Compared with PA projection, AP projection was associated with higher odds of detecting consolidation (aOR 3.27; 95% CI 3.07–3.48), infiltration (aOR 1.90; 95% CI 1.84–1.96), pleural effusion (aOR 1.66; 95% CI 1.60–1.72), atelectasis (aOR 1.63; 95% CI 1.57–1.70), and cardiomegaly (aOR 1.19; 95% CI 1.10–1.28). All effect estimates were consistently above 1, indicating a higher likelihood of these findings in AP compared with PA projections. The strongest association was observed for consolidation.

**Figure 2:**
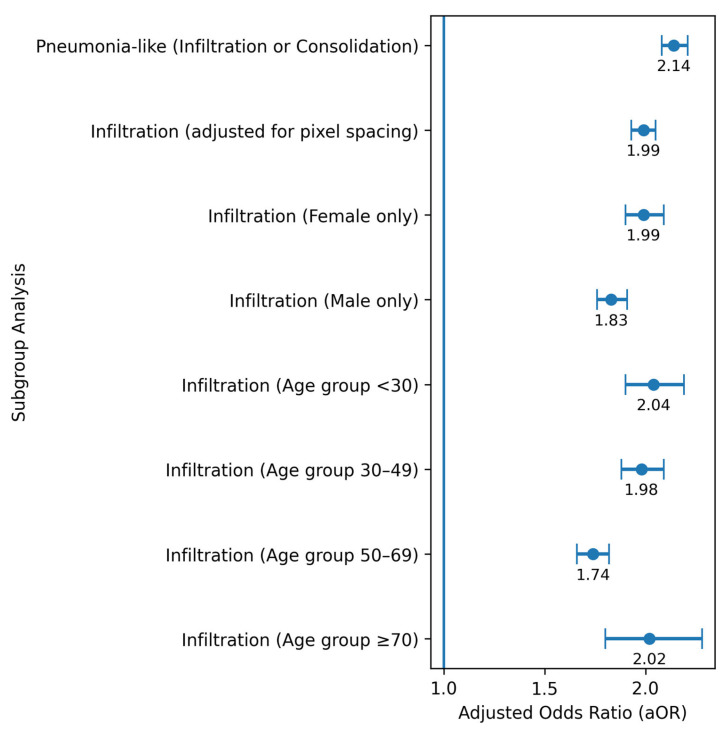
Forest plot illustrating the association between projection type (AP vs. PA) and the detection of pulmonary findings across subgroups. Adjusted odds ratios (aOR) and 95% confidence intervals (CI) are shown for infiltration and the exploratory “pneumonia-like” outcome across multiple subgroups, including age categories, sex, and models adjusted for pixel spacing. To improve interpretability, point estimates are displayed with corresponding confidence intervals and aligned along a common axis. The vertical dashed line indicates the null effect (aOR = 1.0). All estimates were derived from logistic regression models adjusted as appropriate.

Subgroup analyses for infiltration demonstrated consistent associations across all examined strata (Table 3, Figure 2). The highest adjusted odds ratios were observed in patients aged <30 years (aOR 2.04; 95% CI 1.90–2.19) and ≥70 years (aOR 2.02; 95% CI 1.80–2.28), while slightly lower estimates were found in patients aged 30–49 years (aOR 1.98) and 50–69 years (aOR 1.74). Overall, effect sizes were relatively consistent across age groups. Sex-specific analyses showed slightly higher odds in females (aOR 1.99; 95% CI 1.90–2.09) compared with males (aOR 1.83; 95% CI 1.76–1.91). Adjustment for pixel spacing yielded similar results (aOR 1.99; 95% CI 1.93–2.05), indicating the robustness of the observed associations. The composite “pneumonia-like” outcome was also associated with AP projection (aOR 2.14; 95% CI 2.08–2.21), although this exploratory endpoint should be interpreted with caution given its limited clinical specificity.

In the interaction model, AP projection remained associated with infiltration (aOR 1.99; 95% CI 1.90–2.09). The interaction between projection type and sex was modest in magnitude (aOR 0.92; 95% CI 0.87–0.98), indicating a difference in effect size between male and female patients (Table 4).

## 4. Discussion

These findings highlight a potentially underappreciated source of variability in chest radiograph interpretation. The observed associations likely reflect a combination of technical imaging factors and differences in disease prevalence between patient populations undergoing AP and PA imaging. Although prior work has described visual differences between AP and PA radiographs, fewer studies have systematically quantified how projection type influences diagnostic labels in large real-world datasets. By explicitly modeling projection as a determinant of radiographic findings, the present analysis addresses an important methodological factor relevant to both clinical interpretation and the development of machine learning models.

In this large-scale analysis of 112,104 chest radiographs from the ChestX-ray14 dataset, AP projection showed higher odds of detecting thoracic abnormalities, including infiltration, consolidation, pleural effusion, atelectasis, and cardiomegaly. These associations were consistent across age groups and sexes and remained robust after adjustment for age and sex. Consolidation demonstrated the strongest association, with more than a threefold increase in the odds of detection in AP compared with PA projection. Given the observational design, these findings should not be interpreted as causal effects of projection type but rather as associations potentially influenced by both imaging-related and clinical factors.

The increased detection of consolidation in AP views is likely multifactorial. Technical factors such as supine positioning, reduced inspiratory effort, and posterior fluid redistribution may enhance the visibility of gravity-dependent opacities on chest radiographs [8,9]. In addition, AP imaging is frequently performed in acutely ill or immobilized patients who may have a higher prevalence of pulmonary pathology, including pneumonia, pulmonary edema, or aspiration [10,11]. These findings are therefore consistent with a combination of imaging-related effects and confounding by the patient’s condition.

Atelectasis and pleural effusions were also more frequently detected in AP images. Supine positioning may promote basal and posterior atelectasis due to reduced lung expansion and diaphragmatic elevation [12,13]. Similarly, pleural effusions may layer posteriorly in supine patients and appear as diffuse opacification rather than the classical meniscus sign observed in upright PA radiographs [11,14]. These projection-related effects are well recognized in intensive care imaging and should be considered when interpreting radiographic findings across different acquisition conditions.

The modest increase in cardiomegaly detection in AP images is consistent with the known magnification effect associated with AP projection. Because the heart is positioned farther from the detector in AP imaging, the cardiac silhouette may appear artificially enlarged, potentially leading to an overestimation of cardiomegaly if projection type is not explicitly considered [15]. This finding likely reflects a technical imaging artifact rather than a true difference in cardiac size.

From a methodological perspective, projection type represents an important source of potential confounding in both clinical radiology and algorithmic image interpretation. Differences in projection may partly reflect underlying patient characteristics, including disease severity and clinical setting, rather than purely technical imaging factors. Consequently, the observed associations should be interpreted with caution, as residual confounding cannot be fully excluded. Ideally, paired AP and PA radiographs from the same patients would be required to disentangle projection-related effects from patient-related differences; however, such data were not available in the present dataset.

These considerations are particularly relevant for artificial intelligence (AI) systems trained on large radiographic datasets. Previous studies have demonstrated that deep learning models may rely on non-diagnostic visual cues—such as acquisition artifacts, medical devices, or projection markers—to infer disease presence, thereby producing spurious associations and reduced generalizability [16,17,18,19]. Reviews of artificial intelligence in radiology have emphasized that biases present in large public imaging datasets may remain undetected during model development and validation [20]. Furthermore, studies on shortcut learning suggest that models may preferentially exploit easily identifiable visual features rather than clinically meaningful disease patterns [21]. In this context, projection type may act as a proxy feature that models inadvertently learn, rather than as a causal determinant of disease.

Sex-related differences in respiratory infections may represent an additional factor influencing radiographic findings. Previous studies have reported differences between men and women in the incidence and clinical presentation of pneumonia and other respiratory tract infections, which may affect the distribution and radiographic appearance of pulmonary infiltrates [22,23]. However, the interaction observed in this study was modest and should be interpreted cautiously.

Dataset composition also plays a critical role in model performance. In datasets such as the ChestX-ray14 dataset, projection type is not randomly distributed but is closely associated with clinical severity and patient mobility. Consequently, machine learning models trained on such datasets may inadvertently learn projection-specific patterns rather than disease-related radiographic features [8].

Several strategies may help mitigate projection-related biases in imaging datasets. Projection type can be incorporated as an explicit variable during model development, or datasets may be balanced using stratified sampling or projection-specific normalization approaches. In addition, diagnostic performance should ideally be reported separately for AP and PA radiographs to ensure that algorithmic accuracy is not disproportionately influenced by projection-related effects [8,19].

Despite these limitations, the large sample size, statistical consistency, and robustness of the findings support the validity of the observed associations. However, these results should be interpreted as descriptive of dataset-level patterns rather than definitive evidence of projection-induced diagnostic differences. Projection type should therefore be considered an important technical and clinical variable influencing the apparent prevalence and detectability of thoracic abnormalities in chest radiography [24].

In summary, AP projection was consistently associated with an increased likelihood of detecting common thoracic abnormalities, particularly consolidation, infiltration, and pleural effusion. These findings are particularly relevant in the context of the increasing integration of artificial intelligence into radiological workflows and clinical decision support systems [25]. To minimize diagnostic bias and ensure reliable model performance, projection type should therefore be explicitly considered in both clinical interpretation and machine learning applications involving chest radiographs.

## 5. Limitations

This study has several limitations that should be acknowledged. First, the analysis was based on a single publicly available dataset, which may introduce dataset-specific biases related to imaging protocols and reporting practices. The radiographic labels in the ChestX-ray14 dataset were generated using natural language processing (NLP) applied to radiology reports rather than manual expert annotation. Although this approach enables large-scale analyses, it may be subject to misclassification and may not fully capture subtle or implicitly described findings. Previous studies have demonstrated variable sensitivity and specificity of these labels, particularly for findings such as infiltration and consolidation. Consequently, some degree of label noise cannot be excluded and may have influenced the observed associations. Second, the dataset lacks detailed clinical metadata, including patient positioning (e.g., supine vs. upright), clinical severity, and the indication for imaging. These factors are closely related to both projection type and disease prevalence and may therefore act as unmeasured confounders. Third, projection information was limited to a binary classification (AP vs. PA), without further differentiation between imaging conditions such as semi-erect positioning, portable radiography, or bedside acquisition, which may differ in technical and diagnostic characteristics. Fourth, due to the retrospective observational design, causal relationships between projection type and the detection of specific radiographic findings cannot be established. The observed associations may partly reflect underlying differences in patient populations rather than purely technical imaging effects. Finally, the dataset originates from a single institution (NIH Clinical Center), which may limit generalizability to other healthcare settings with different imaging protocols, equipment, and patient populations. Despite multiple subgroup and sensitivity analyses, residual confounding cannot be fully excluded, particularly with respect to disease severity, patient immobility, and comorbidity burden. Despite these limitations, the large sample size, consistency of findings across subgroups, and robustness of the observed associations support the validity of the results. Future studies using manually annotated datasets and incorporating detailed clinical metadata are warranted to further clarify projection-related variability in chest radiography.

## 6. Conclusions

In this large-scale analysis of 112,104 chest radiographs from the ChestX-ray14 dataset, AP projection showed consistently higher odds of detecting common thoracic abnormalities—including infiltration, consolidation, pleural effusion, atelectasis, and cardiomegaly—compared with PA projection. These associations remained robust across age and sex strata and persisted after adjustment for key covariates. These findings indicate that projection type represents an important technical and clinical factor influencing the apparent prevalence and detectability of radiographic findings. Importantly, projection may also act as a source of dataset bias, reflecting both imaging-related effects and differences in underlying patient populations. These associations should be interpreted with caution, as they likely reflect a combination of technical imaging factors and residual confounding related to the patient’s condition and clinical context. Accounting for projection type is therefore essential to improve the accuracy of clinical interpretation and to ensure the robustness and generalizability of artificial intelligence models trained on large imaging datasets. Future studies should validate these findings using expert-annotated datasets and incorporate detailed clinical and positional metadata to better disentangle projection-related effects from true disease prevalence and underlying disease severity.

## Figures and Tables

**Table 1:** Baseline characteristics of chest radiograph cases by projection type. Posteroanterior (PA) and anteroposterior (AP) projections were compared with respect to sex and age distribution. Sex is reported as absolute numbers with percentages. Age is summarized as mean ± standard deviation (SD), median with interquartile range (IQR), and minimum–maximum values.

Variable	PA (n = 67,299) (%)	AP (n = 44,805) (%)
Male sex, n (%)	37,149 (55.2)	26,211 (58.5)
Female sex, n (%)	30,150 (44.8)	18,594 (41.5)
Age, mean ± SD (years)	47.3 ± 16.2	46.2 ± 17.2
Age, median (IQR), years (min–max)	49 (36–59)	48 (33–59)
Age, range (min–max), years	1–95	1–94

**Table 2:** Adjusted odds ratios (aOR) for radiographic findings by projection type. Logistic regression models were used to estimate the association between projection type (anteroposterior [AP] vs. posteroanterior [PA]) and the presence of specific radiographic findings. All models were adjusted for age (per year) and sex (male vs. female). Results are presented as adjusted odds ratios (aOR) with 95% confidence intervals (CI) to emphasize effect size and precision of the estimates. An aOR greater than 1 indicates a higher likelihood of detecting the respective finding in AP compared with PA projections.

Finding	aOR (AP vs. PA)	95% CI
Consolidation	3.27	3.07–3.48
Infiltration	1.90	1.84–1.96
Pleural effusion	1.66	1.60–1.72
Atelectasis	1.63	1.57–1.70
Cardiomegaly	1.19	1.10–1.28

**Table 3:** Subgroup and sensitivity analyses: impact of projection on infiltration detection. Logistic regression models were used to assess the association between anteroposterior (AP) vs. posteroanterior (PA) projection and the likelihood of detecting pulmonary infiltration. Subgroup analyses were performed across age categories (<30, 30–49, 50–69, ≥70 years) and by sex (male only, female only). A sensitivity analysis was conducted with adjustment for pixel spacing. In addition, a combined endpoint labeled “pneumonia-like” (presence of either infiltration or consolidation) was evaluated. This composite endpoint was included for exploratory purposes and should be interpreted with caution. All models were adjusted for age and/or sex as appropriate. Results are presented as adjusted odds ratios (aOR) with 95% confidence intervals (CI) to emphasize the effect size and precision of the estimates.

Analysis	aOR (AP vs. PA)	95% CI
Age < 30 years	2.04	1.90–2.19
Age 30–49 years	1.98	1.88–2.09
Age 50–69 years	1.74	1.66–1.82
Age ≥ 70 years	2.02	1.80–2.28
Male	1.83	1.76–1.91
Female	1.99	1.90–2.09
Adjusted for pixel spacing	1.99	1.93–2.05
Pneumonia-like outcome *	2.14	2.08–2.21

* Exploratory endpoint; interpret with caution.

**Table 4:** Interaction analysis: effect modification by sex on the association between projection and infiltration. A logistic regression model was used to examine whether the association between projection type (AP vs. PA) and the likelihood of detecting pulmonary infiltration differed by sex. The model included an interaction term (projection × sex) and was adjusted for patient age. Results are reported as adjusted odds ratios (aOR) with 95% confidence intervals (CI) to emphasize effect size and precision of the estimates. The interaction term reflects differences in the association between projection type and infiltration across sex subgroups and should be interpreted as a measure of effect modification rather than a causal interaction.

Variable	aOR	95% CI
Projection (AP vs. PA)	1.99	1.90–2.09
Sex (Male vs. Female)	1.07	1.03–1.12
Projection × Sex (interaction)	0.92	0.87–0.98

## Data Availability

The NIH ChestX-ray14 dataset used in this study is publicly available via the NIH repository: https://nihcc.app.box.com/v/ChestXray-NIHCC (accessed on 15 March 2026). All analyses can be reproduced using this publicly accessible dataset.
